# Extirpation of the Entire Thyroid Gland

**Published:** 1867-03

**Authors:** E. L. Marshall

**Affiliations:** Keithsburg, Mercer Co., Ill.


### CHICAGO MEDICAL JOURNAL.

VOL. XXIV. MARCH, 1867. No. 3.

### ORIGINAL CONTRIBUTIONS.

*Extirpation of the Entire Thyroid Gland.* By E. L. MARSHALL, M.D., Keithsburg, Mercer Co., Ill.

In your Journal for December last, I find the report of a case of removal of one-half of the thyroid gland, by Prof. Wm. Warren Greene, being the third instance (so far as my own information extends) of this operation having been successfully performed in America. Dr. Geo. McClellan, Sr., of Philadelphia, is said to have succeeded in a single instance, (his being a single lobe only,) and my own, performed Jan. 18th, 1852, comprising the entire gland, with Dr. Greene's more recent case, of August 19th, 1866; all of which patients have had good recoveries. I am unaware, at this time, of there being a single case of a fatal or unsuccessful operation in this country.

In view of the anatomical relations concerned in this operation, it is not surprising that leading men of our profession should be slow to recognize the propriety of any attempt at ablation of that body. Prof. Sam'l D. Gross says:—"Thus, whether we view this operation in relation to the difficulties which must necessarily attend its operation, or with reference to the severity of the subsequent inflammation, it is equally deserving of rebuke and condemnation. No honest and sensible surgeon, it seems to me, would ever engage in it." And that daring surgeon, Prof. Mott, in his lectures, was accustomed to speak of the operation in about the same emphatic terms.

In Europe, we find that the operation has been had in quite a number of recorded cases, a fair proportion of which seem to have resulted favorably. It is nothing very surprising that, in the time of Albucasis, a patient should have died of hemorrhage, or that the young lady mentioned by Palfin succumbed during the operation, from the same cause; that one of the patients cited by Gooch perished, exhausted, at the end of eight days, and that the other was saved by assistants, who relieved each other for the space of a week, in order to compress without intermission, by means of their fingers, all the arterial branches which had been opened; though an officer, whose history is given by Percy, also perished from hemorrhage, and that the operation performed by Dupuytren resulted fatally in thirty-five hours. The instances of that operation, by the French surgeons Freytag, Theden, Desault, Giraudi, and Fodere may teach us something of the hazards of the operation, still, I cannot fully endorse the sweeping terms of condemnation with which many of our eminent writers have sought to discourage all attempts to ablate this gland. Since, in contradistinction to the unfortunate cases referred to above, we have the fact that of the French surgeons, Peckel and Ravaton performed it with success in several instances, and Vogel has been no less fortunate. Græfe succeeded in saving two of his three patients. Indeed, I think it safe to say that no greater proportion than one to three of our reported cases have perished during, or from the effects of this operation. Is it not true that the operation is more generally condemned from the fact that it is so seldom necessary, expedient, or justifiable, than for the reason that it is more hazardous than many other of our capital operations—as amputation at the hip-joint, exsection of the knee and ankle-joints, trephining cranial bones, hysteriotomy, ovariotomy, etc., etc.? If so, we must then confront the question, as to whether the operation ever becomes necessary, from the circumstances investing the case and condition of the patient. Dr. Warren Greene tells us that his patient suffered greatly from pressure upon the organs of respiration; "was unable to lie down; required constant watching, during hours of broken sleep, lest she should die of suffocation; that she suffered much from headache and giddiness, and that she could not stoop without losing her consciousness." All of these symptoms had been gradually increasing, and for the last two months, an almost daily aggravation had been noticeable." Dr. Greene does not inform us as to whether this case had been submitted to judicious treatment prior to his observation, on the 19th of August, 1866, but in the absence of such history, we must infer that his patient, Mrs. Klopf, had availed herself of the best obtainable medical advice, and that she had been subjected to such treatment as is usually successful in the management of goitre.

Again, he says, that "Profs. Ford, Palmer, and Storer, and Drs. Smith, Brewster, and others, examined her; and all agreed that a few days, at most, would terminate her existence, while the thread was liable to snap at any hour." Dr. Greene has furnished us with a strong case—one that will be twice read before the most cautious of our surgeon friends would be willing to assert that Dr. Greene was not justifiable in giving to his patient the last and only resource left to her through our profession.

I have taken the liberty of referring to Dr. Greene's case the more in detail, for the reason that it is a parallel one to that of which I now propose to give a history.

My patient, Mr. John Mank, now residing at Bridger's Corners, Mercer Co., Ill., had consulted a number of eminent surgeons, among whom were Profs. Mussey and Joseph N. McDowell, also Prof. Brainard, and S. S. Cooper, of Peoria, all of whom, after an examination of the case, gave it as their opinion, that an operation for removal of the gland might, eventually, become justifiable. To my own knowledge, Mr. Mank has been, from time to time, through a period of many years, subjected to vigorous treatment, with reference to absorption of the tumor, all of which had not even checked the progress of the morbid growth.

Referring to my notes, I find that on the 12th of January, 1852, the patient had for three months been unable to lie down, and required constant watching to save him from suffocation; was subject to spasms of great dyspnœa; constant headache; had twice had convulsions from attempts to lie down; was mentally despondent, declaring that he preferred death to such an existence. After making a full statement of the hazards incidental to extirpation, it being the only resource left upon which to fall back or recommend, I left it with himself for decision. He promptly requested me to undertake the removal of the tumor. In this decision I had the happiness of having the concurrence of Drs. Adam Clandaning and A. B. Campbell, whose valuable counsel and assistance I here take the occasion to acknowledge.

The patient, Mr. Mank, aged 40, a gentleman of high order of intelligence, and, previous to his health having been impaired by his present affliction, a man of great muscular power, possessing a high order of moral courage—such courage, indeed, as I have seldom, if ever, witnessed in any other person.

The patient, declining the use of an anæsthetic, took his position, (agreeable to his own request, in an arm-chair,) and I proceeded to operate, by making an incision through the median line, commencing a few lines above, and completing as much below, the lower border of the tumor. This wound I transformed into a crucial incision; detached the flaps and dissected them down to their base; fleshy fibres that were not easily pushed aside were divided transversely, until the entire superficial surface of the body was exposed. I now separated the tumor from its bed, by dividing the several fasciæ on the director and tearing up the areolar tissue with the finger and handle of the scalpel, until the base of the gland was freely exposed. There was but little hemorrhage from the superficial vessels of the tumor, and none that required especial care, save a couple of small arteries that were found lying alongside the thyro-hyoid muscle, imbedded in the cellular lamella. These were ligated—they were, perhaps, branches of the lingual or maxillary arteries. The pedicle of the tumor being reached, I

found each lobe to be supplied with two arteries (the superior and inferior thyroid). Each portion containing a vessel was surrounded by a heavy ligature of loosely twisted saddlers' silk. The inferior arteries were now sealed, without any disturbance of organic function; but on attempting to tighten the ligatures of the superior vessels, we were met by a difficulty that was well-nigh fatal to our operation. This occurred incidental, as I believe, to pressure upon a branch of the great sympathetic, the pneumo-gastric, the glosso-pharyngeal, or fibres from two, or possibly all three, of those important nerves. Be that as it may, on any attempt to ligate those vessels, the patient suffered from severe paroxysmal cough, labored breathing, and a lessening of the force of the heart's action that was truly alarming. As we could not hope to be able, under the circumstances, to find and exclude the small, thread-like fibre from the ligature, we did that which I believed to be the next best thing in the premises—to destroy the functional integrity of the nerve; this was done by tightening the cord and holding it firmly so long as the patient was able to endure and live, when it was loosened and the interrupted organic functions were allowed to resume something of their normal condition. This required but a few minutes, and here was witnessed a heroism upon the part of Mr. Mank that was truly sublime. Nothing daunted by the peril, so well understood by himself, he was always ready, after his brief rests, to request that the work of nerve-crushing might be resumed.

After several efforts of this kind, I had the happiness of knowing that the difficulty was so far overcome as to admit of ligation, which was accordingly done, and the tumor was removed without farther trouble.

The wound was closed by interrupted sutures, and the patient was placed in bed, he having retained his position, as first placed, for one hour and twenty minutes. He seemed to suffer less from shock than almost any other person upon whom it has been my fortune to witness the performance of any severe surgical operations. The pulse 86, somewhat depressed; voice and breathing better than before the operation; was cheerful, remarking to his medical friends, "gentlemen, give to yourselves no uneasiness on my account, for I shall recover." A full anodyne was given; water-dressing to the wound; and the room to be kept quiet.

*Jan. 17th, 8 A.M.* Found that Mr. Mank has had snatches of sleep during the night; has been awakened repeatedly by the occurrence of spasmodic cough; is beginning to expectorate a thick, tenacious mucus; pulse 95; some headache. Directed a continuation of morphia at regular intervals, with a reference to an arrest of pulmonic irritation. *9 P.M.* Has spent a comfortable day. Directed gruel, as before, with anodyne sufficient to allay cough; mucilage for drink.

*18th, 8 A.M.* Patient passed a bad night, in consequence of cough and labored respiration. Inspiration 40 per minute; pulse 102; some headache. Ordered sal. Epsom, ℥j.; morphiæ sulph., gr. j.; spt. nitrici dulc., ℥ss.; aquæ menth. ℥ij.:—dose ℥ss. at two hour intervals. *9 P.M.* Bowels have been freely moved. Expectorating freely; cough troublesome; no headache; skin moist; cheerful.

*19th, A.M* Has had hurried breathing while sleeping; pulse 106, compressible; appetite better; asks for other diet. Ordered potato soup and beef-tea; aconite with morphia. *P.M.* Pulse 106; inspirations 40; skin moist; tongue slightly coated with lightish fur; bowels have been freely moved during the day; has spasmodic cough, at which time large quantities of mucus are expectorated.

*20th, A.M.* Has spent a better night; symptoms unchanged. Ordered tinct. digitalis, in place of aconite, with which exception, treatment continued. *P.M.* No change in symptoms or treatment.

*21st, A.M.* Pulse 96, soft, and compressible; cough less troublesome; expectorates freely; breathing better; inspirations 30 per minute; skin moist; bowels have moved freely; urinary secretion normal; tongue as before; appetite good. Ordered chicken-water, with cracker crumbs. Digitalis continued, with tinct. ferri mur.

*22d, A.M.* Symptoms favorable; pulse 85. Treatment continued.

From this time forward, until the *25th*, nothing occurred of any special interest, either in point of symptoms or of treatment. The general management was, as before indicated, rather aimed at correcting troublesome symptoms, than to any preconceived plan of medication. The water-dressings were continued. On the evening of the 25th day, during an unusually severe paroxysm of coughing, a sudden gush of arterial blood announced the fact of secondary hemorrhage from one of the large vessels of the pedicle; this was immediately controlled by the nurse, who had been previously instructed how to act in case of such emergency. On seeing the patient, I applied an astringent powder, suggested by Prof. McClellan, Sr., Philadelphia, consisting of tannic acid, pulv. gallici, and g. acaciæ. This, together with small pledgets of lint, used as a compress, served to control the bleeding perfectly, the wound now suppurating kindly and fast filling up with granulations.

No farther accident marring the progress of cure, the condition of the patient steadily improved; the ligatures had all come away, without farther hemorrhage, by the fifteenth day; and on the twenty-fifth day from the date of the operation, Mr. Mank returned home to his residence, some twenty-five miles distant in the country, the wound being almost entirely closed, and he able to walk without much fatigue.

The tumor, when examined, was found to be hard, firm, with cartilaginous deposits, and weighed one pound eleven ounces, avoirdupois.

I would especially invite attention to the fact that, in the c se of Mr. Mank, the only serious difficulty encountered during the operation, was in the dissection of the nervous communication between the gland and nervous centres, while in Dr. Greene's case, no such trouble was had, neither have I any knowledge of any such barriers to the successful execution of the operation being encountered by any other party attempting the extirpation of this gland; while all refer to the fearful hemorrhage incidental to the rupture of delicate superficial veins, spread like a network over the body. In my own case, the walls of those vessels were sufficiently firm to admit of careful dissection, without risk to their continuity.

*Query.*—Was this case simply one of anomalous distribution of nerve fibre, or have my professional friends been more fortunate, in having excluded them from their ligature? I believe that the former proposition is the correct one, since I have not touched a cadaver since that time, in which I have not made search for nerve filaments in that part, and, as yet, have been unable to trace a single filament into that body.

In conclusion, I would observe that the patient is still living and in the enjoyment of almost perfect health, having scarcely had a day of illness since his recovery from the loss of his entire "thyroid gland," now fifteen years since, showing conclusively that the functions of that body are not indispensable to the healthy performance of other organic functions.

